# Calculation of Hole Concentrations in Zn Doped GaAs Nanowires

**DOI:** 10.3390/nano10122524

**Published:** 2020-12-16

**Authors:** Jonas Johansson, Masoomeh Ghasemi, Sudhakar Sivakumar, Kilian Mergenthaler, Axel R. Persson, Wondwosen Metaferia, Martin H. Magnusson

**Affiliations:** 1 Solid State Physics, Lund University, Box 118, 221 00 Lund, Sweden; sudhakar.sivakumar@ftf.lth.se (S.S.); mergenthaler.kilian@gmail.com (K.M.); wondwosen.metaferia@nrel.gov (W.M.); martin.magnusson@ftf.lth.se (M.H.M.); 2 NanoLund, Lund University, 221 00 Lund, Sweden; 3 Thermo-Calc Software AB, 169 67 Solna, Sweden; gh.phys@gmail.com; 4 nCHREM/Centre for Analysis and Synthesis, Lund University, Box 124, 221 00 Lund, Sweden; axel.persson@liu.se

**Keywords:** nanowires, vapor-liquid-solid growth, impurity doping

## Abstract

We have previously demonstrated that we can grow p-type GaAs nanowires using Zn doping during gold catalyzed growth with aerotaxy. In this investigation, we show how to calculate the hole concentrations in such nanowires. We base the calculations on the Zhang–Northrup defect formation energy. Using density functional theory, we calculate the energy of the defect, a Zn atom on a Ga site, using a supercell approach. The chemical potentials of Zn and Ga in the liquid catalyst particle are calculated from a thermodynamically assessed database including Au, Zn, Ga, and As. These quantities together with the chemical potential of the carriers enable us to calculate the hole concentration in the nanowires self-consistently. We validate our theoretical results against aerotaxy grown GaAs nanowires where we have varied the hole concentration by varying the Zn/Ga ratio in the aerotaxy growth.

## 1. Introduction

Semiconductor nanowires have proven to be promising building blocks in a wide variety of applications, including life sciences [1], electronics [2], photonics [3], solid state lighting [4], and energy harvesting [5]. Most of these applications rely on the electronic properties of the nanowires, and for this reason, it is of the highest importance to be able to control their conductivity. In semiconductor materials, this is done by doping, which means that point defects which add electrons or holes are introduced in a controlled way.

The most common method to grow semiconductor nanowires is by means of vapor-liquid-solid (VLS) growth, where a liquid seed particle, often regarded as a catalyst, is used to confine the growth, resulting in a one-dimensional morphology [6,7]. There are numerous investigations on the doping of nanowires during VLS growth [8,9]. In this case, the dopants reach the growth interface through the catalyst particle, which both poses limitations and possibilities. Thermodynamic effects can influence this kind of doping. The solubility of the dopant in the catalyst particle should for instance be sufficiently high and when the dopants have entered the particle, they must not alter the chemical potentials of the semiconductor reactant species in such a way that the driving force for growth ceases. Nevertheless, in several materials systems the dopant atoms are soluble in the particle during VLS growth, and dopant atoms incorporate in the nanowires during growth.

Doping during VLS growth is not yet well understood. However, there are a few attempts to explain certain aspects and to model dopant incorporation in nanowires during growth. Schwalbach and Voorhees [10] have proposed a thermodynamic model, which includes the fluxes of dopants and wire reactant species through the particle. While their model provides general understanding of the doping process, it is hard to validate since the necessary thermodynamic parameters are often unknown and the dopant flux through the particle is difficult to estimate. Hijazi et al. [11] have modeled the amphoteric behavior of Si in Au-catalyzed GaAs nanowires. Their model, which has similarities with the Hannay model [12], considers the full thermodynamic description of the catalyst particle and allows investigating effects of changing the state of the particle on the doping. For instance, it was found that increasing the As concentration in the particle favors n-type doping, which shows that it is possible to tune the amphoteric behavior of Si in GaAs nanowires by altering the growth conditions [11]. In addition, the chemical potential of As is known to oscillate during growth due to depletion effects correlated with monolayer nucleation and growth. This As depletion has been found to have a strong effect on the Si doping of GaAs nanowires grown by the VLS mechanism [13]. Moreover, Dubrovskii et al. [14] have developed a model based on growth thermodynamics where the doping level of VLS grown nanowires is related to the doping level of a thin film grown from the vapor phase under corresponding conditions.

In this investigation, we demonstrate calculations of doping concentrations in VLS grown nanowires from first principles, without using any free parameters. The materials system that we consider is Zn doped, Au-catalyzed GaAs nanowires and we base our calculations on the Zhang–Northrup model for the defect formation energy [15]. We compare our calculated hole concentrations with measured hole concentrations of aerotaxy [16,17] grown nanowires where we have varied the Zn/Ga ratio in the gas phase.

## 2. Materials and Methods

### 2.1. Density Functional Theory Calculations

We have performed density functional theory (DFT) calculations to study Zn substitution on a Ga site in GaAs with zinc blende crystal structure using the supercell method with Abinit code [18]. Supercells of 8, 16, 64, and 128 atoms were used to fully relax the strain (both with respect to cell shape and volume) due to the defect. The 8-atom supercell was chosen as the conventional unit cell, the 16-atom supercell is composed of 8 primitive unit cells in a 2 × 2 × 2 arrangement. The 64-atom supercell is composed of 8 conventional unit cells in a 2 × 2 × 2 arrangement, while the 128-atom supercell is built from 64 primitive unit cells in a 4 × 4 × 4 arrangement. We used the projector augmented-wave (PAW) method to describe the interaction of electrons with the ionic cores [19]. The generalized gradient approximation (GGA) was used for the exchange-correlation functional [20]. The first Brillouin zone integration was performed over a k-point mesh of between 65 and 85 k-points using the Monkhorst–Pack method [21]. The wave functions were expanded in a plane-wave basis set with the cutoff energy of 680 eV. A broadening factor of 0.136 eV was used to facilitate the convergence of the total energies. The convergence energy was chosen as 0.01 eV/atom, which is the chemical accuracy. The total energy of this structure was calculated using the converged input variables. The convergence parameters were fixed for the largest supercell and then we used the same set of parameters for the smaller systems. We have also calculated the total energy of the non-defected GaAs with zinc blende structure, Zn with hexagonal close-packed structure, and Ga with orthorhombic structure with the same input values.

### 2.2. Calculation of Chemical Potentials

The chemical potentials of Zn and Ga in the liquid quaternary alloy catalyst particle containing Zn, Ga, As, and Au were calculated using the software Thermo-Calc [22]. The thermodynamic data were extrapolated from the As-Ga-Zn ternary system [23] and the As-Ga [24], As-Zn [23], As-Au [25], Au-Ga [26], Au-Zn [27], and Ga-Zn [28] binary systems. When using Thermo-Calc’s graphical interface for chemical potential calculations, a reference state has to be chosen and we choose the enthalpy of the stable state at 298 K and 1 bar as reference state for each species. Before we applied the resulting chemical potentials, we replaced this reference state with the total energy of the stable state of each species from our DFT calculations. These reference states are equivalent and the reason for the replacement is to be consistent with the calculation of the energy difference between the defect and the perfect supercells.

### 2.3. Aerotaxy Growth

The GaAs nanowires in this study were grown with the newly developed method of aerotaxy, which allows mass-production of nanowire material at relatively low cost [17]. In aerotaxy, the nanowires grow directly in the gas phase from Au seed particles, and the growth rate is on the order of 1 µm/s, which is at least 100 times faster than in metalorganic vapor phase epitaxy (MOVPE). The high growth rate is mainly attributed to the high partial pressures of the growth precursors, around 1 mbar, which are again orders of magnitude higher than in MOVPE.

The seed particles were formed as an aerosol by evaporation of Au at 1800 °C, rapid cooling, and size selection to 80 ± 20 nm diameter in a differential mobility analyzer [29]. The Au aerosol was fed into the aerotaxy reactor [17], where nanowire growth occurred at a temperature of 550 °C and at atmospheric pressure. The growth precursors used were trimethylgallium (TMGa), introduced together with the Au aerosol (with a flow of 850 sccm), and arsine (AsH_3_), injected together with the sheath gas (with a flow of 2500 sccm), see ref. [17] for details. The partial pressures of the precursors are defined as the volume flow of precursor divided by the sum of aerosol and sheath flows, and were $p_{\mathrm{TMGa}}$ = 2.77 mbar and $p_{{\mathrm{AsH}}_{3}}$ = 1.23 mbar. For the Zn doping, diethylzinc (DEZn) was mixed in together with the TMGa, and the dopant content in the gas phase is characterized by the fraction $p_{\mathrm{DEZn}}/p_{\mathrm{TMGa}}$. After growth, the nanowires were collected on Si substrates for further study by means of an electrostatic precipitator [30].

### 2.4. Doping Characterization

The aerotaxy nanowires were characterized by both low temperature micro-photoluminescence (µ-PL) spectroscopy and basic field effect (FE) mobility measurements. For µ-PL characterization, the Si substrate carrying the nanowires was bonded to a cryostat with colloidal Ag paste. The cryostat and the sample were cooled using liquid helium down to approximately 4 K. A frequency doubled solid-state laser emitting at 532 nm excited the samples. The measured excitation power density was about 200 W/cm^2^. The µ-PL setup cannot resolve single nanowires, so the measured signal originates from a randomly oriented nanowire ensemble of roughly 100 wires. The hole concentration for the Zn doped GaAs nanowires was calculated using the relationship $p={\left(2m_{h}^{*}\Delta E_{p}\right)}^{3/2}/\left(3\pi^{2}\hbar^{3}\right)$, which can be derived from the integral of the density of hole states times the Fermi-Dirac distribution, similar to the approach in ref. [31]. The parameter $m_{h}^{*}$ is the effective mass of holes, given by $m_{h}^{*}=0.53m_{0}$, where $m_{0}$ is the resting mass of the electron and ℏ is the reduced Planck’s constant. The parameter $\Delta E_{p}$ is the difference in full-width-at-half-maximum of the PL peak of the doped sample and the PL peak of the undoped sample.

**Figure 1:**
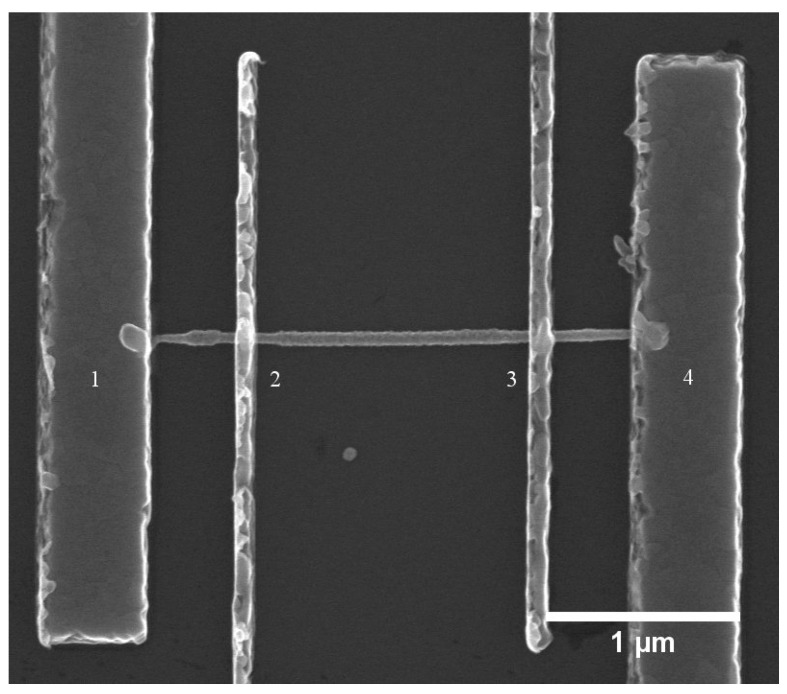
SEM image of a Zn doped GaAs nanowire with interdigitated metal electrodes. Four-probe resistivity and contact resistance measurements are carried out by passing a current through probes 1 and 4 while measuring the voltage difference between probes 2 and 3.

For single wire FE measurements, the nanowires were mechanically transferred onto a Si substrate with a lithographically pre-defined substrate map on top of a 110 nm (100 nm SiO_2_ and 10 nm HfO_2_) oxide layer; the map enables us to locate and contact individual nanowires on the substrate. After wire transfer, the substrate surface was imaged using a scanning electron microscope (SEM, Hitachi SU8010 FE SEM, Hitachi High-Technologies Corporation, Tokyo, Japan) and suitable single nanowires were identified for contacting using metal electrodes. The substrate was then spin coated with PMMA 950 A6 to form the lift-off layer. The nanowires were contacted by interdigitated Ti/Au (10/180 nm) metal electrodes defined by electron beam lithography while the substrate acted as a back gate. The device design was optimized for four-probe resistivity measurements by making the midsection (between electrodes 2 and 3) relatively long as shown in Figure 1. For nanowire FE measurements, a constant source (1)–drain (4) voltage was applied over the nanowire in order to study the transfer characteristics of the nanowire field effect transistor (FET). The back gate voltage was swept from −5 to 5 V and back in steps of 0.5 V. The mobility and the carrier concentration can be extracted from the transfer characteristics of the nanowire FET.

### 2.5. Catalyst Particle Composition

The catalyst particle compositions of the nanowires were systematically investigated using a Jeol 3000F transmission electron microscope (TEM, Jeol 3000F, Jeol, Tokyo, Japan), operated at 300 kV in scanning mode. Compositional analysis was performed using the TEM’s X-ray energy dispersive spectroscopy (EDS) detector from Oxford Instruments (Abingdon, Oxfordshire, UK) and quantified using its corresponding analysis software (Inca). After cool-down, the particles had segregated into multiple compositional phases and compositional analysis was performed over the particle, as a whole, to get the total composition. For each sample, six nanowires were investigated.

## 3. Theoretical Model

Zinc is known to form shallow, singly charged acceptors in GaAs by substituting Ga atoms [32]. High doping levels, above 10^20^ cm^−3^, can be achieved and the acceptor state can be regarded as uncompensated [33]. In order to describe the hole concentration, p, in GaAs resulting from Zn substitution on Ga lattice sites, we use the defect formation energy, as proposed by Zhang and Northrup [15]. According to this formalism, the formation energy of a defect, X, in charge state q is given by

(1) $$E_{f}\left[X^{q}\right]=E_{tot}\left[X^{q}\right]-E_{tot}\left[\mathrm{perfect}\right]-\;\underset{i}{\sum }n_{i}\mu_{i}+q\varepsilon_{F},$$

where $E_{tot}\left[X^{q}\right]$ is the total energy of a supercell containing the defect, $E_{tot}\left[\mathrm{perfect}\right]$ is the total energy of a perfect cell without the defect, $n_{i}$ are the number of atoms of type i that have been added ($n_{i}>0$) or removed ($n_{i}<0$) from the supercell when creating the defect, and $\mu_{i}$ are the respective chemical potentials. Finally, $\varepsilon_{F}$ is the Fermi level, measured from the valence band edge. For our case of Zn doping in GaAs, the formation energy is given by

(2) $$E_{f}\left[{\mathrm{Zn}}^{-1}\right]=E_{tot}\left[{\mathrm{Zn}}^{-1}\right]-E_{tot}\left[\mathrm{GaAs}\right]-\mu_{\mathrm{Zn}}+\mu_{\mathrm{Ga}}-\varepsilon_{F}.$$

Since the reference states of the chemical potentials are the total energies of Zn and Ga in their reference states, we write the chemical potential of these atomic species in the liquid state as $\mu_{i}=E_{i}+\delta \mu_{i}$, where $E_{i}$ is the total energy of i in its reference state (hexagonal close packing for Zn and orthorhombic for Ga) and $\delta \mu_{i}$ is the remaining, temperature and composition dependent part of the chemical potential. For the sake of convenience, we rewrite Equation (2) as

(3) $$E_{f}=\Delta E-\delta \mu_{\mathrm{Zn}}+\delta \mu_{\mathrm{Ga}}-\varepsilon_{F},$$

where $\Delta E=E_{tot}\left[{\mathrm{Zn}}^{-1}\right]-E_{tot}\left[\mathrm{GaAs}\right]-E_{\mathrm{Zn}}+E_{\mathrm{Ga}}$. Now we can express the concentration of Zn in GaAs,

(4) $$\left[{\mathrm{Zn}}^{-1}\right]=Ne^{-\frac{E_{f}}{k_{B}T}},$$

where N is the number of sites per volume available for Zn substitution, $k_{B}$ is Boltzmann’s constant, and T is the absolute temperature. As Zn replaces Ga in GaAs, which has the zincblende crystal structure, $N=4/a^{3}$, where a is the lattice constant of GaAs.

The Fermi level and the hole concentration, p, which is the property of interest, are related through [34]

(5) $$p=4\pi {\left(\frac{2m_{h}^{*}k_{B}T}{h^{2}}\right)}^{3/2}\overset{\infty }{\underset{0}{\int }}\frac{\sqrt{\xi }d\xi }{1+e^{\xi +\varepsilon_{F}/\left(k_{B}T\right)}},$$

where $m_{h}^{*}$ is the effective mass of holes given by $0.53m_{0}$, with $m_{0}$ the resting mass of the electron and h is Planck’s constant. Next, we need to consider charge neutrality in the doped nanowire and this condition can be expressed as

(6) $$p+N_{D}^{+}=n+N_{A}^{-},$$

where $N_{D}^{+}$ is the concentration of positively charged donors, n is the electron concentration, and $N_{A}^{-}$ is the concentration of negatively charged acceptors. The hole and electron concentrations are connected through the law of mass action, $pn=n_{i}^{2}$, where $n_{i}$ is the intrinsic carrier concentration. In our case, the Zn doping can be considered uncompensated, so we can set $N_{D}^{+}=0$ and n will be orders of magnitude smaller than p. This leads to the particularly simple case,

(7) $$p=\left[{\mathrm{Zn}}^{-1}\right].$$

Now we can calculate p by combining Equations (3)–(5), and (7) and solve the equations self-consistently. In the case of light p-doping it is possible to approximate Equation (5) by the Boltzmann distribution, $p=N_{V}e^{-\varepsilon_{F}/\left(k_{B}T\right)}$, where $N_{V}$ is the effective density of states in the valence band [34]. Combining this with Equations (3), (4), and (7) will result in a closed form equation for p. Since our materials are heavily, and even degenerately, doped we use the Fermi–Dirac integral in Equation (5) in our calculations.

Most importantly, all calculations of the doping concentration have to be accompanied by a calculation of

(8) $$\Delta \mu_{\mathrm{GaAs}}=\mu_{\mathrm{Ga}}+\mu_{\mathrm{As}}-\mu_{\mathrm{GaAs}},$$

which is the driving force for GaAs formation. This quantity has to be positive in order for GaAs to form. If $\Delta \mu_{\mathrm{GaAs}}<0$ for the considered parameters, the calculated doping concentration lacks physical significance. Since the nanowires are quite thick, with diameters on the order of 100 nm, we neglect any finite size effects on the defect incorporation.

Finally, a generalizing remark: if additional charged defects contribute to the carrier concentration, we need to consider one formation energy each for all of them in order to express their concentrations. A similar self-consistent approach can be used to solve for the carrier concentration. The charge neutrality condition will however be more complicated in this case. Concerning carriers, electrons or holes can be considered, whichever gives the simplest calculations. This is because they are linked through the law of mass action.

## 4. Results and Discussion

In Table 1 we show the results from the DFT calculations where we have calculated the total energies $E_{tot}\left[{\mathrm{Zn}}^{-1}\right]$ and $E_{tot}\left[\mathrm{GaAs}\right]$ for supercells of varying size. Now we need to extrapolate the energy difference $E_{tot}\left[{\mathrm{Zn}}^{-1}\right]-E_{tot}\left[\mathrm{GaAs}\right]$ for N→∞, that is for an isolated impurity. This we do by fitting the energy difference to a linear function, a+b/N, according to a truncated Makov-Payne expansion [35]. The data in Table 1 fit well to this line and we extract the intercept, a, which represents the energy difference at infinite dilution to $E_{tot}\left[{\mathrm{Zn}}^{-1}\right]-E_{tot}\left[\mathrm{GaAs}\right]$ = 402.7191 eV. We have also calculated the total energy per atom of Zn in its hexagonal close packed phase, $E_{\mathrm{Zn}}=-1755.7219$ eV and of Ga in its orthorombic phase, $E_{\mathrm{Ga}}=-2157.9088$ eV. This results in ΔE = 0.5321 eV.

Next, we calculate the p-doping concentrations based on the theoretical model outlined in the previous section. We start doing this for generic values of the temperature and catalyst particle compositions in order to study how the doping concentration changes with these parameters. Then we compare our model calculations with measured doping concentrations of aerotaxy grown gold alloy catalyzed GaAs nanowires.

**Figure 2:**
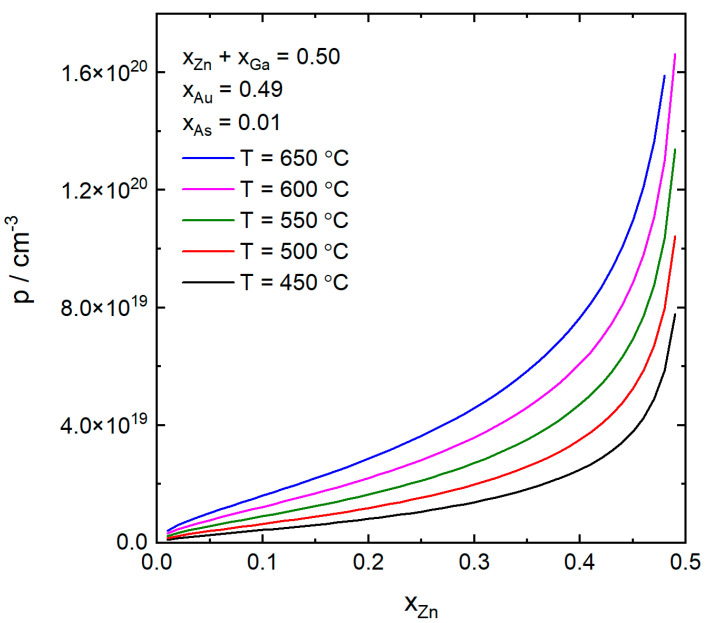
The p-doping concentration as a function of the atomic fraction of Zn in the catalyst particle at various temperatures, from 450 to 650 °C. The atomic fractions of As and Au were kept constant at $x_{\mathrm{As}}=0.01$ and $x_{\mathrm{Au}}=0.49$. The atomic fraction of Zn was varied at the expense of Ga so that $x_{\mathrm{Zn}}+x_{\mathrm{Ga}}=0.50$.

In Figure 2, we show how the p-doping concentration, p, changes with the atomic fraction of Zn in the particle, $x_{\mathrm{Zn}}$, for different values of the temperature and for constant atomic fractions of Au and As. We observe that increasing the Zn at the expense of Ga increases p from the 10^18^ cm^−3^ range up to the 10^20^ cm^−3^ range. For low to mid values of $x_{\mathrm{Zn}}$ the increase is approximately linear but as the Zn is further increased the increase in p is stronger. Increasing the temperature also increases p, but not as strongly as by increasing the Zn concentration. The reason that the 650 °C curve stops at lower $x_{\mathrm{Zn}}$ than the lower temperature curves is that at higher $x_{\mathrm{Zn}}$, and thus lower $x_{\mathrm{Ga}}$, the driving force for GaAs formation, according to Equation (8), is no longer positive. That is, $\Delta \mu_{\mathrm{GaAs}}<0$ at these conditions. Since the solubility increases with temperature, this effect gets more and more pronounced the higher the temperature.

**Figure 3:**
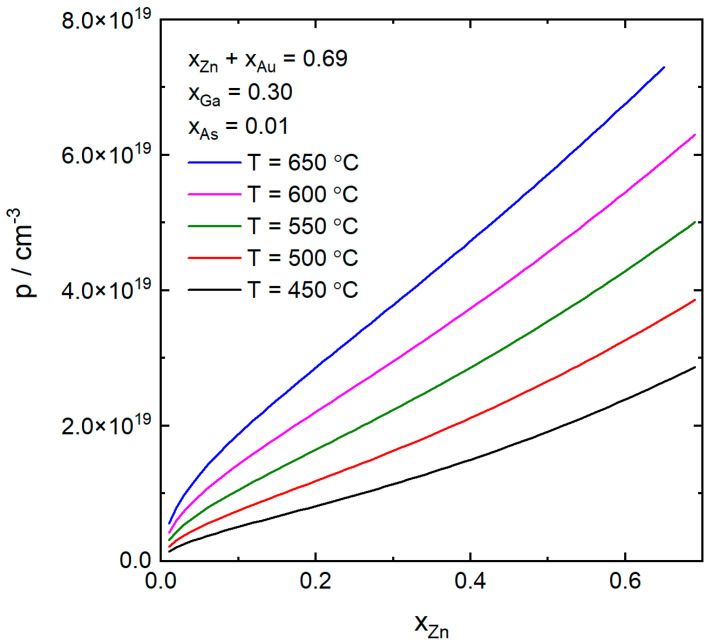
The p-doping concentration as a function of the atomic fraction of Zn in the catalyst particle at the same temperatures as in Figure 2. The atomic fractions of As and Ga were kept constant at $x_{\mathrm{As}}=0.01$ and $x_{\mathrm{Ga}}=0.30$. The atomic fraction of Zn was varied at the expense of Au so that $x_{\mathrm{Zn}}+x_{\mathrm{Au}}=0.69$.

In Figure 3, we show the p-doping concentration as a function of $x_{\mathrm{Zn}}$ for the same temperatures as in the previous case. Here the As and the Ga concentrations are held constant and the concentration of Zn is varied at the expense of the Au concentration so that $x_{\mathrm{Zn}}+x_{\mathrm{Au}}=0.69$. In this case, where the Au instead of the Ga is varied, the increase in p as a function of $x_{\mathrm{Zn}}$ is smaller than in the previous case (Figure 2) and the increase is stronger for low Zn concentrations, also in contrast to the previous case. Also, here, for the 650 °C case, $\Delta \mu_{\mathrm{GaAs}}$ becomes negative at a lower $x_{\mathrm{Zn}}$ than for the lower temperature cases.

**Figure 4:**
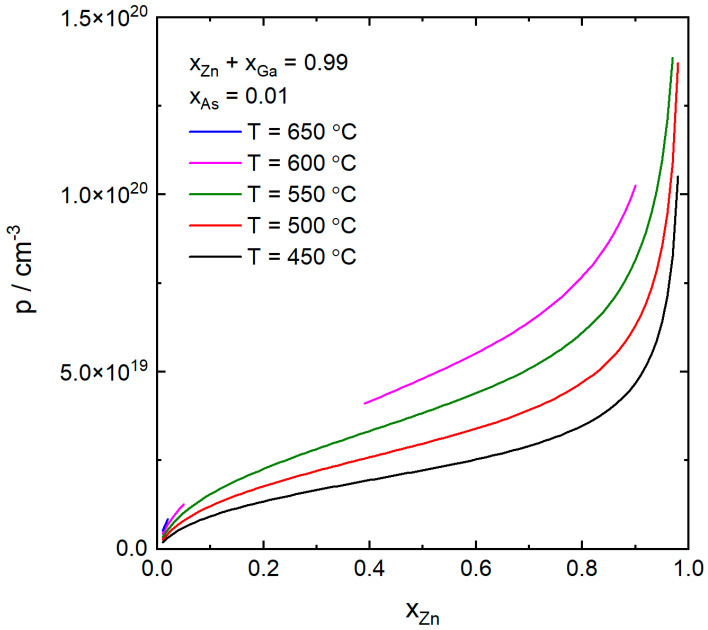
The p-doping concentration as a function of the atomic fraction of Zn in the catalyst particle at the same temperatures as in Figure 2 for self-catalyzed growth, that is $x_{\mathrm{Au}}=0$. The atomic fraction of As was kept constant at $x_{\mathrm{As}}=0.01$ and the atomic fraction of Zn was varied at the expense of Ga so that $x_{\mathrm{Zn}}+x_{\mathrm{Ga}}=0.99$.

**Figure 5:**
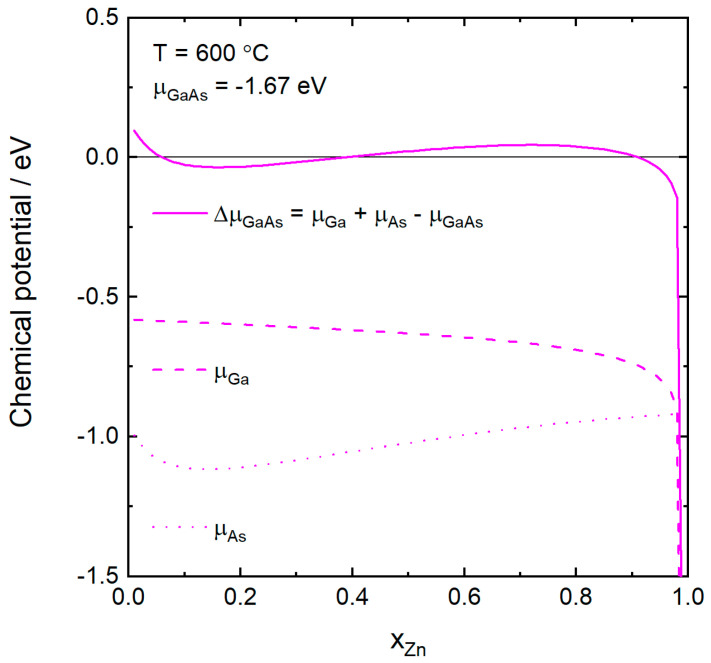
The chemical potentials of As, Ga, and the chemical potential difference for GaAs, $\Delta \mu_{\mathrm{GaAs}}$, as a function of the atomic fraction of Zn in the catalyst particle at 600 °C. The chemical potential of GaAs, which is composition independent, is at this temperature −1.67 eV. Only when $\Delta \mu_{\mathrm{GaAs}}>0$ can GaAs grow, and this occurs for $x_{\mathrm{Zn}}\leq 0.05$ and $0.39\leq x_{\mathrm{Zn}}\leq 0.90$.

In Figure 4, we investigate the case of self-catalyzed growth. Here only the As concentration is constant and p is shown as a function of $x_{\mathrm{Zn}}$ for the same temperatures as previously. The curves are only shown if the corresponding $\Delta \mu_{\mathrm{GaAs}}$ is positive, that is if GaAs can form. For the three lower temperatures, the Zn concentration can be varied over a wide interval and the p-doping concentration increases strongly with $x_{\mathrm{Zn}}$, especially for high values of $x_{\mathrm{Zn}}$, similar to Figure 2. At the highest temperature, 650 °C, we see that the Zn concentration has to be less than about 0.02 for GaAs to form. Higher values of Zn might instead lead to the formation of other solid phases, such as zinc arsenide. At 600 °C, p as a function of $x_{\mathrm{Zn}}$ exhibits a discontinuous behavior, where growth of Zn doped GaAs is possible only if $x_{\mathrm{Zn}}\leq 0.05$ or $0.39\leq x_{\mathrm{Zn}}\leq 0.90$. The explanation for the discontinuity is the non-linearity of $\Delta \mu_{\mathrm{GaAs}}$ as a function of $x_{\mathrm{Zn}}$, which is illustrated in Figure 5. Here we see that $\Delta \mu_{\mathrm{GaAs}}>0$ only if $x_{\mathrm{Zn}}\leq 0.05$ or $0.39\leq x_{\mathrm{Zn}}\leq 0.90$. For other values of $x_{\mathrm{Zn}}$, the catalyst particle is undersaturated ($\Delta \mu_{\mathrm{GaAs}}<0$) and GaAs cannot form. By increasing the As concentration, $\mu_{\mathrm{As}}$ would increase and $\Delta \mu_{\mathrm{GaAs}}$ would be positive for a larger and eventually continuous set of Zn concentrations, such as for the 450–550 °C curves in Figure 4.

We have also investigated how the p-doping concentration varies with the As concentration in the catalyst particle. We set $x_{\mathrm{Zn}}=0.20$, $x_{\mathrm{Ga}}=0.30$, and $x_{\mathrm{As}}+x_{\mathrm{Au}}=0.50$ and varied $x_{\mathrm{As}}$ from 0.001 to 0.05 for temperatures from 450–650 °C to find an increase in p of only about 10–15%. That is, the doping concentration is quite insensitive to the As concentration.

Now, after investigating these trends we turn to comparison with experiments. We have grown Au particle catalyzed, Zn doped GaAs nanowires using the aerotaxy process. In these experiments the temperature was constant at 550 °C but the nominal gas phase ratio of Zn/Ga (the pressure of DEZn divided by the pressure of TMGa in the reaction chamber) was varied from 0.025 to 0.6. The shape of the nanowires is similar to what was reported by Yang et al. [36]. In the current investigation, we vary the Zn fraction in the gas phase over a much broader interval than what was done in the prior investigation [36].

**Figure 6:**
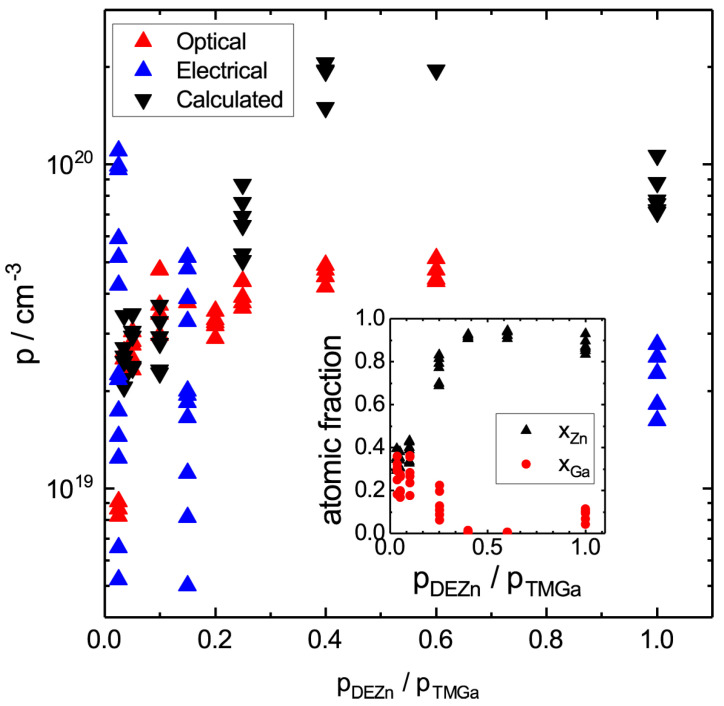
The p-doping concentration as a function of the nominal gas phase ratio of Zn and Ga precursors, $p_{\mathrm{DEZn}}/p_{\mathrm{TMGa}}$, during aerotaxy growth. The red and blue triangles account for optically and electrically measured doping concentrations, respectively. The black downward pointing triangles represent calculated doping concentrations based on the atomic fractions measured by EDS and shown in the inset, $x_{\mathrm{As}}=0.01$, $x_{\mathrm{Au}}=1-x_{\mathrm{As}}-x_{\mathrm{Ga}}-x_{\mathrm{Zn}}$, and T=550 °C.

The p-doping concentrations in the nanowires were extracted from optical and electrical measurements and are shown as red and blue triangles in Figure 6. The p-doping concentrations were also calculated using the model developed here. As input values we used the concentrations of Ga, Zn, and Au in the catalyst particle as measured after growth. For each growth condition, six nanowire catalyst particles were examined, and the atomic fractions of Zn and Ga are shown in the inset of Figure 6. The rest is Au and As. However, no As was detected, since its concentration is below the detection limit of EDS (about 1 at%). Since the solidified catalyst particles are phase segregated, these concentrations are averaged over the entire particles. Since the cool down to room temperature is fast for aerotaxy, the measured concentrations are good estimates of the concentrations during nanowire growth.

Since no As was detected after growth, we set the As atomic fraction to 0.01, which is the detection limit of EDS and thus an upper limit. The three other atomic fractions were rescaled so that all the four atomic fractions sum to one. Using these four atomic fractions and the growth temperature as input, we calculated the chemical potentials of Zn, As, and Ga in the liquid catalyst particle and of GaAs in its solid zincblende phase. The chemical potentials were then used to calculate the p-doping concentration and to verify that there is a driving force for GaAs growth.

When comparing the calculated and the measured p-doping concentrations, we see that there is a reasonable match given that no fitting parameters were used and that the measured p-concentrations are so scattered. Especially so, since for doping evaluations the main interest is often the order of magnitude and typically not the exact concentrations.

The two lowest groups of calculated values in Figure 6 show a very good overlap with the measured values, while the data for $p_{\mathrm{DEZn}}/p_{\mathrm{TMGa}}$ of 0.4 and 0.6 diverge. In these two cases, the calculations were based on very low concentrations of Ga, which can be seen in the inset. With such low concentrations of Ga, growth of GaAs is on the border of being possible. In fact, for several of these conditions $\Delta \mu_{\mathrm{GaAs}}<0$, which is the reason for the few data points here (since we only plot the calculated data points for which $\Delta \mu_{\mathrm{GaAs}}>0$). It is possible and perhaps even likely that in these cases the catalyst particle was segregated also during growth with growth occurring from a part of the catalyst with less Zn and more Ga, which would lead to nanowires with lower p.

Also, in the $p_{\mathrm{DEZn}}/p_{\mathrm{TMGa}}=1.0$ case, the calculated p-concentration is higher than the electrically measured one, indicating that also in this case, the growth concentration of Ga could have been higher and the growth concentration of Zn could have been lower than what is indicated in the inset. This would lead to lower doping p-doping concentrations, approaching the measured ones. It is interesting to note that the $p_{\mathrm{DEZn}}/p_{\mathrm{TMGa}}$ = 0.4 and 0.6 cases give the on average highest measured doping concentrations and that the measured doping concentration decreases when increasing $p_{\mathrm{DEZn}}/p_{\mathrm{TMGa}}$ to 1.0. One should however be cautious since we here compare two different measurement techniques. However, the calculated p-concentrations follow the same trend, even if they are higher than the measured ones.

## 5. Conclusions

We have demonstrated how to calculate doping concentrations of vapor–liquid–solid grown nanowires using a combination of DFT and thermodynamic calculations. Such calculations are of great advantage when controlling and predicting the doping concentrations for a wide variety of experimental conditions.

Our calculations compare well with experimental hole concentrations in Zn-doped GaAs nanowires grown by aerotaxy. In these calculations, we did not use any fitting parameters. The As concentration in the catalyst particle is indeed unknown and was set to a small value (1 at%), but the doping concentration is in any case quite insensitive to the As concentration. Finally, it is interesting to note that the calculations confirm our experimental observation that it is difficult to achieve low and intermediate doping levels. Except for very low Zn concentrations in the catalyst particle and low growth temperatures, the hole concentration in the nanowire tends to be high (around 10^19^ cm^−3^ and higher).

## Figures and Tables

**Table 1:** The total energy of an *N* atoms large GaAs supercell containing one Zn atom on a Ga site and the difference in total energy of two *N* atoms large GaAs supercells, one containing one Zn atom on a Ga site and the other one defect free GaAs.

N	$E_{tot}\left[Zn^{-1}\right]/\mathrm{eV}$	$E_{tot}\left[Zn^{-1}\right]-E_{tot}\left[\mathrm{GaAs}\right]/\mathrm{eV}$
8	−8939.9832	402.9457
16	−18,283.0257	402.8363
64	−74,340.6705	402.7707
128	−149,084.1712	402.7106
